# Real-Time Temperature Sensing Using a Ratiometric Dual Fluorescent Protein Biosensor

**DOI:** 10.3390/bios13030338

**Published:** 2023-03-03

**Authors:** Alanna E. Sorenson, Patrick M. Schaeffer

**Affiliations:** Molecular and Cell Biology, College of Public Health, Medical and Veterinary Sciences, James Cook University, Douglas, QLD 4811, Australia

**Keywords:** GFP, mCherry, ratiometric, DSF-GTP, fluorescent, thermal, temperature, differential scanning fluorimetry, DFPTB

## Abstract

Accurate temperature control within biological and chemical reaction samples and instrument calibration are essential to the diagnostic, pharmaceutical and chemical industries. This is particularly challenging for microlitre-scale reactions typically used in real-time PCR applications and differential scanning fluorometry. Here, we describe the development of a simple, inexpensive ratiometric dual fluorescent protein temperature biosensor (DFPTB). A combination of cycle three green fluorescent protein and a monomeric red fluorescent protein enabled the quantification of relative temperature changes and the identification of temperature discrepancies across a wide temperature range of 4–70 °C. The maximal sensitivity of 6.7% °C^−1^ and precision of 0.1 °C were achieved in a biologically relevant temperature range of 25–42 °C in standard phosphate-buffered saline conditions at a pH of 7.2. Good temperature sensitivity was achieved in a variety of biological buffers and pH ranging from 4.8 to 9.1. The DFPTB can be used in either purified or mixed bacteria-encapsulated formats, paving the way for in vitro and in vivo applications for topologically precise temperature measurements.

## 1. Introduction

Temperature control is critical to physical, chemical and biological experiments. Monitoring sample temperature is highly desirable but requires the use of secondary measuring equipment. The real-time monitoring of temperature in a sample is often impractical, particularly when the reaction volumes are relatively small [1,2,3,4,5,6,7]. Individual fluorescent proteins have previously been used as temperature biosensors [8,9,10]. However, since fluorescence intensity is concentration-dependent, an accurate knowledge of biosensor concentration and standardization is required for precise temperature determination with a single biosensor [11]. The use of ratiometric comparison of fluorescence emission has been applied to address this problem through the use of dual probes with different [12,13] or identical excitation wavelengths [14,15]. Ratiometric-based biosensors also enable internal calibration while reducing the impact of excitation intensity, spatial dispersion, and sample autofluorescence on temperature sensing [16].

Various different types of ratiometric-based temperature sensors have been developed, with different responses to increasing temperature including; one fluorophore with constant fluorescence paired with another that either increases or decreases in fluorescence, both fluorophores either increasing or decreasing in fluorescence at different rates or one fluorophore with increasing fluorescence while the other has decreasing fluorescence [16]. Of these temperature biosensors, those that pair fluorophores with opposite responses to increasing temperature have the greatest sensitivity and resolution [16]. All existing ratiometric temperature sensors of this type are of synthetic rather than biological construction [17,18,19,20,21,22], often requiring relatively complex synthesis steps and operation in non-aqueous solvents, precluding the ability to be used in biological samples. A biological ratiometric optical thermometer with comparable capabilities would enable both in vivo and in vitro applications, including the ability for the development of genetically encoded temperature sensors.

Herein, we describe the design and evaluation of a simple, inexpensive and sensitive ratiometric dual fluorescent protein temperature biosensor (DFPTB). The intensity ratio between cycle three green fluorescent protein (uvGFP) and monomeric red fluorescent protein (mCherry) fluorescence emissions in response to increasing temperature was evaluated using multiple formats and different instruments. The DFPTB was then used to measure relative changes in temperature reliably and reproducibly in various real-time thermal cycler applications. The data demonstrate the utility of the DFPTB in identifying non-uniformity in real-time cycler thermal blocks, evaluating the accuracy of their temperature gradients and detecting deviations in experimental sample volume. The DFPTB exhibited superior temperature sensitivity in comparison to other fluorescent thermosensors [16,17] and could be applied in a mixed bacteria-encapsulated format without need for purification. Additionally, we discuss how the DFPTB could be improved for both intracellular and absolute temperature measurement.

## 2. Materials and Methods

### 2.1. Protein Expression and Purification

The GFP cloning vector was produced as described previously [23]. The pcDNA3 mCherry LIC cloning vector (6B) was a gift from Scott Gradia (Addgene plasmid # 30125). The mCherry coding sequence was amplified from pcDNA3 mCherry LIC cloning vector (6B) using the primers psJCU400 forward: 55″-GGATCCGGCGGTGATATACTAGCTGCCATCATCAAGGAG-33″ and psJCU401 reverse: 55″-GAATTCTTAGGACTTGTACAGCTCGTCCATGCCGC-33″. The PCR product was digested with BamHI and EcoRI and ligated into identically cut differential scanning fluorimetry (DSF-GTP) expression vector, pIM013 [23], to create pAC282. GFP and mCherry were expressed in *E. coli* BL21(DE3)RIPL in Terrific broth with 100 µg/mL ampicillin and 50 µg/mL chloramphenicol via autoinduction. In 1L culture flasks, 100 mL media with 4 mM glucose and 0.4 mM galactose was inoculated with a full inoculation loop of bacteria scraped from an overnight Luria broth agar with 100 µg/mL ampicillin and 50 µg/mL chloramphenicol master plate. The cultures were incubated at 37 °C with shaking at 250 RPM until log phase was reached, then reduced to 16 °C for 72 h. Lysis and purification was performed as described previously [24]. Following ammonium sulphate precipitation, GFP and mCherry were resuspended in phosphate-buffered saline (PBS), pH 7.2. Purity was assessed by SDS-PAGE and quantification performed using Bradford assay.

### 2.2. General Differential Scanning Fluorimetry (DSF) Assays

Reactions were performed with 1 µM (unless otherwise stated) each of uvGFP and mCherry in 50 µL aliquots of varying buffers in low profile skirted 96-well PCR plates (Bio-Rad, South Granville, Australia) with plate sealing film (Bio-Rad). All reactions were performed in at least triplicate. Melt curve analysis was performed in a Bio-Rad CFX-96 with an initial 2 min RT equilibration followed by melt curves of varying dwell times (1, 10, 30 and 60 s) from 4–90 °C, using the FAM (_ex_450–490 nm, _em_510–530 nm) and Texas Red (_ex_560–590 nm, _em_610–650 nm) channels. Buffers tested included: PBS (pH 7.2); 50 mM phosphate (pH 7.8) with 10% glycerol; 50 mM HEPES (pH 7.5) with 10% glycerol; 50 mM citrate (pH 6.0) with 10% glycerol; 50 mM ammonium sulphate (pH 5.7) with 10% glycerol; 50 mM phosphate (pH 7.8) with 300 mM NaCl, 10% glycerol, 20 mM imidazole and 2 mM β-mercaptoethanol; PBS (pH 7.2) with 1% BSA; and PBS (pH 7.2) with 4% BSA and 0.005% Tween. pH stability was assessed in 50 mM phosphate buffer (pH 4.7–9.1). Raw data were extracted using CFX Maestro software (Bio-Rad).

### 2.3. Sensitivity to Temperature Change and Resolution

For the detection of sensitivity to changing temperature, DSF assays were performed as described previously (Section 2.2) with uvGFP and mCherry in PBS, pH 7.2 (Sigma, Macquarie, Australia) with a 30 s dwell time. Dual relative fluorescence (RFU) was recorded for 30 s at staggered isothermal temperatures with 10 °C increases and decreases from 10–70 °C, returning to 4 °C between each change. For the assessment of resolution and precision, DSF was performed as described in Section 2.2, with RFU measurement for 10 s at staggered isothermal incubations with 0.1–0.2 °C increases from 37–38 °C, returning to 4 °C between each change. Additionally, melt curves were performed from 4–70 °C with a 10 s dwell time at 0.1 °C increments. For all procedures (unless otherwise stated) fluorescence data were background-subtracted. Normalized fluorescence (NF) data for each fluorophore were determined by dividing by the RFU_n_ value at a given temperature by the initial RFU_i_ value at 4 °C (NF = RFU_n_/RFU_i_). The ratio data of normalized RFU (Ratio = NF_uvGFP_/NF_mCherry_) were calculated and plotted against the temperature. Absolute sensitivity (S_a_) was calculated according to the equation: S_a_ = [(δ(NF_uvGFP_/NF_mCherry_)/δT [17,21].

### 2.4. Relative Temperature Quantification

DSF assays were performed as described previously (Section 2.3). Dual relative fluorescence (RFU) was recorded for 30 s at various temperatures (37, 42, 50 and 65 °C) following equilibration at RT for 2 min. Relative fluorescence for each fluorophore was normalized as described previously (Section 2.3). The relationship between the natural log (ln) of the normalized ratio (Ratio = NF_uvGFP_/NF_mCherry_) and temperature from both melt curves and isothermal temperature readings were fit with a linear regression model and used to interpolate observed temperature using each standard curve.

### 2.5. Temperature and Gradient Uniformity

DSF assays were performed as described previously (Section 2.4). Dual RFU was recorded as described previously at isothermal temperatures (12, 25, 37, 42, 50 and 65 °C). Dual RFU was also recorded across temperature gradients of 30–50 °C and 50–70 °C. Each gradient was performed vertically across a full 96-well plate with three repeats. Standard curves were produced with the general DSF assay melt curve and isothermal curves with 30 s dwell time. Normalization and transformation were performed as described previously (Section 2.4). Observed temperatures were calculated from individual well standard curves and the whole plate average.

### 2.6. Intermachine Variability

DSF assays were performed as described previously (Section 2.5). Dual RFU was recorded on appropriate channels for the detection of uvGFP and mCherry on an additional Bio-Rad CFX 96 and a Bio-Rad Opus 96 at another site. Melt curves and isothermal temperature holds were performed with the same settings. Normalization and observed temperature calculation were performed as described previously (Section 2.4). Gradient reproducibility was compared as described previously (Section 2.5).

### 2.7. In Vivo Temperature Sensing in Mixed Bacteria-Encapsulated Format

Single colonies of BL21(DE3)RIPL *E. coli* cells expressing uvGFP and mCherry were resuspended in various buffers, mixed and diluted to obtain RFU readings comparable to 1 µM protein suspensions, then subjected to two consecutive DSF runs with 2 min equilibration at 4 °C and melt curves from 4–70 °C with 0.5 °C increments and 10 s dwell time. Purified uvGFP and mCherry were run in the same buffers in tandem as control. Buffers tested included: PBS (pH 7.2); 50 mM phosphate (pH 4.8); 50 mM phosphate (pH 7.8) with 300 mM NaCl, 10% glycerol, 20 mM imidazole and 2 mM β-mercaptoethanol; and 50 mM phosphate (pH 7.8) with 1.4 M NaCl, 10% glycerol, 20 mM imidazole and 2 mM β-mercaptoethanol. Normalization and transformation were performed as described previously (Section 2.4).

### 2.8. Minimum DFPTB Concentration and Detection of Volume Discrepancy

For the determination of minimum DFPTB concentration, a typical DFPTB suspension containing 1 µM of each protein was subjected to serial 2-fold dilutions. For the detection of volume discrepancy between wells, standard DSF assays with 50 µL DFPTB were performed alongside DFPTB aliquots at 25 and 75 µL in triplicate. Normalization and transformation were performed as described previously (Section 2.4). DSF assays were performed as described previously (Section 2.5).

### 2.9. Absorbance and Fluorescence Spectra

Temperature-dependent absorbance and fluorescence spectra were measured with a Shimadzu UV2600 and RF6000 spectrometer, respectively. Constant-Temperature Cell Holders (P/N 202-30858-44 (UV2600) and 206-24930-41/42/58 (RF6000)) were connected in parallel using a Huber KISS E circulator to heat the samples (room temperature, 37, 40, 51 and 67 °C). The sample temperature was confirmed using a thermometer in the UV2600 reference cell prior to scanning. Each protein was tested separately in a Hellma Suprasil Quartz Ultra-Micro Cell Cuvette in PBS, pH 7.2 with uvGFP at 50 µM and mCherry at 2.5 µM. UV–Vis absorption spectra were recorded from 900–200 nm for both proteins. Fluorescence spectra were collected in dilute solutions (absorbance = 0.1 at the excitation wavelength) to avoid concentration quenching and excimer emission. For uvGFP, the emission spectra were recorded from 465–650 nm and excited at 460 nm. For mCherry, the emission spectra were recorded from 575–800 nm and excited at 570 nm.

## 3. Results and Discussion

**Figure 1:**
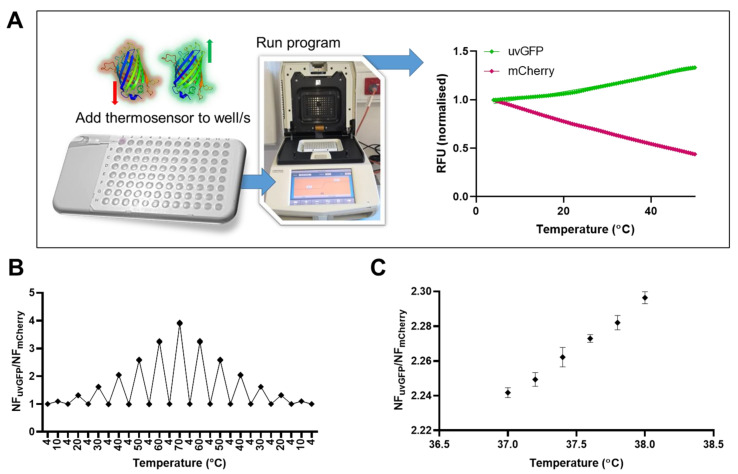
(**A**) DFPTB workflow and typical temperature-dependent change in normalized fluorescence for uvGFP (NF_uvGFP_ = RFU_n_/RFU_i_) and mCherry (NF_mCherry_ = RFU_n_/RFU_i_). (**B**) Ratio of normalized fluorescence (NF_uvGFP_/NF_mCherry_) demonstrates reproducible sensitivity to temperature change between 4–70 °C. Background subtraction was not applied before normalization to highlight the thermal stability of DFPTB. (**C**) Resolution in the physiological range (37–38 °C) at 0.2 °C increments with temperature returning to 4 °C between each temperature change. Fluorescence data are background-subtracted and normalized for highest resolution. Error bars indicate standard deviation (*n* = 3). Data were analyzed using the Kruskal–Wallis test (*p* = 0.0073). Pairwise comparison of changes in temperature between 0.2 °C increments were analyzed using paired *t*-tests (*p* values ranged from 0.0075–0.1117).

The accuracy of a thermal block may influence the outcome of PCR and DNA melt curves. Well-to-well consistency of the temperature across a thermal block is paramount for reproducible PCR [25], DNA melt curves [25,26,27], DSF [28] and DSF-GTP data [24,29]. As such, it is crucial that real-time thermal cyclers achieve a set temperature as accurately and consistently as possible across the entire thermal block. Fluorescence emission decay at a set wavelength upon increasing temperature has been demonstrated previously for mCherry [14] as has the opposite response for wtGFP [10], which is similar to the uvGFP used in this study (Q80R/F99S/M153T/V163A). However, the combination of two fluorescent proteins, uvGFP and mCherry, with opposite responses to temperature acting as a ratiometric thermosensor is novel. Our workflow for the assessment of relative temperature change in real-time thermocyclers was straightforward (Figure 1), comprising a simple mixing of mCherry and uvGFP in a suitable buffer, addition to a 96-well plate in the desired wells, sealing and running the required program using appropriate channels (typically Green/FAM and Red/Texas Red).

In response to increasing temperature, uvGFP demonstrated a consistent fractional increase in fluorescence and mCherry a consistent decrease (Figure 1A). The temperature range from 4–70 °C resulted in an exponential relationship of the ratio of normalized fluorescence (NF_uvGFP_/NF_mCherry_) as a function of temperature (Figure S1A). The DFPTB demonstrated excellent stability, sensitivity, reproducibility and reversibility to changing temperature in the 4–70 °C range (Figure 1B). Advantageously, changes in mCherry and uvGFP concentrations as well as their proportion have no significant effect on the ratiometric relationship (Figure S1B). This relationship is also unaffected by incubation time (Figure S1C). The DFPTB demonstrated sub-degree resolution in the physiological range (37–38 °C) with a precision of 0.2 °C (Figure 1C and Figure S1D).

The performance of the DFPTB was further evaluated in different buffer systems. The best sensitivity and accuracy were achieved in 50 mM phosphate buffer, pH 7.8 with 1.4 M NaCl, 10% glycerol, 20 mM imidazole and 2 mM β-mercaptoethanol (Table 1). However, the DFPTB can be used in a variety of buffers and pH ranging from 4.8–9.1 (Figure S2 and Table 1). Generally, the DFPTB was functional within the stability range (dependent on pH and ionic strength parameters) of uvGFP and mCherry with a safe maximum temperature of 70 °C due to the relatively high transition midpoint of unfolding (T_m_) of these proteins (T_m_ ~80 °C) [23,24,29,30,31]. However, buffer pH < 6.0 reduced the working temperature range to 4–45 °C (Figure S2 and Table 1), with some minimal reductions in buffers at pH = 6.0 (Figure S2 and Table 1). Overall, the DFPTB was useful in most biologically relevant buffers in the physiological temperature range of 25–42 °C, where it performed with an absolute sensitivity up to 6.7% °C^−1^ (Table 1).

Of note, other comparable synthetic dual fluorescent temperature sensors require solubilization in solvents, such as methylcyclohexane [17], dimethylformamide [18], dimethyl sulfoxide [19], isopropanol [19], dichloromethane [20] and ethanol/glycerol [21], limiting their application in vivo. Of these, only the frustrated static excimers [17] were also demonstrated to be temperature sensitive in a biologically relevant buffer system (PBS) in the physiological range; however, they would not be practical for intracellular applications.

Many fluorescent temperature sensors have sensitivities lower than 2% °C^−1^, which makes them unsuitable for applications requiring sub-degree resolution [16]. However, the relatively high sensitivity of the DFPTB is on par with or superior to other ratiometric fluorescent temperature sensors (Table S1). Indeed, the DFPTB demonstrated high precision in the physiological range (37–38 °C) of 0.2 °C in melt curves (Figure S1D) and isothermal incubations (Figure 1C). This degree of precision is not limited to this narrow temperature window. Indeed, such small temperature differences are detectable from 25–55 °C (Figure S1D). For static temperature measurement, ~0.2 °C is the limit of precision achievable in real-time thermal cyclers, due to signal fluctuation in this configuration (systematic pairwise comparison of changes in temperature between 0.2 °C increments produced *p* values of 0.0075–0.1117). However, for continuous temperature monitoring, the resolution of 0.1 °C (*p* values of 0.003–0.0483) was achievable in single wells (Figure S1D–F). Such precision is required for potential application in in vivo temperature sensing where sub-degree resolution is essential.

**Figure 2:**
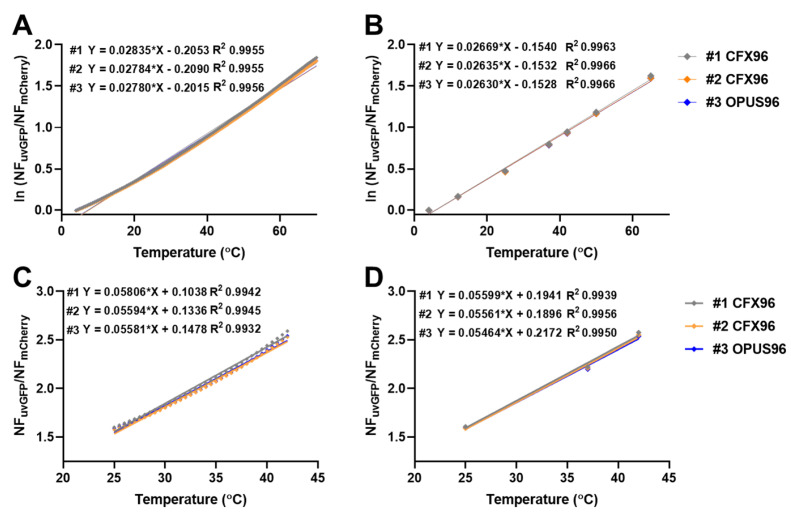
Temperature agreement between three different instruments. (**A**) Natural log-transformed ratio values for temperature range 4–70 °C. Data are averages for a 96-well plate. (**B**) Natural log-transformed ratio values for isothermal incubations at specified temperatures (4, 12, 25, 37, 42, 50 and 65 °C). Data are averages obtained for a 96-well plate. (**C**,**D**) Ratio values in the pseudolinear range 25–42 °C for melt curves and isothermal holds, respectively. Melt curves were performed with 0.5 °C intervals for 30 s and isothermal incubations were held for 30 s.

The DFPTB was evaluated with three different real-time thermocyclers, located at different sites (a Bio-Rad CFX96 at one site and another CFX96 as well as a Bio-Rad OPUS 96 at a second site). Both melt curve and isothermal temperature runs yielded near identical slopes in good agreement between comparable programs and mathematical transformations (Figure 2A–D). Furthermore, very little difference could be observed between temperature determination using standard curves obtained from an individual well (data not shown) or the entire plate average for either melt curves (Figure 2A,C) or isothermal temperature incubation (Figure 2B,D), respectively.

**Figure 3:**
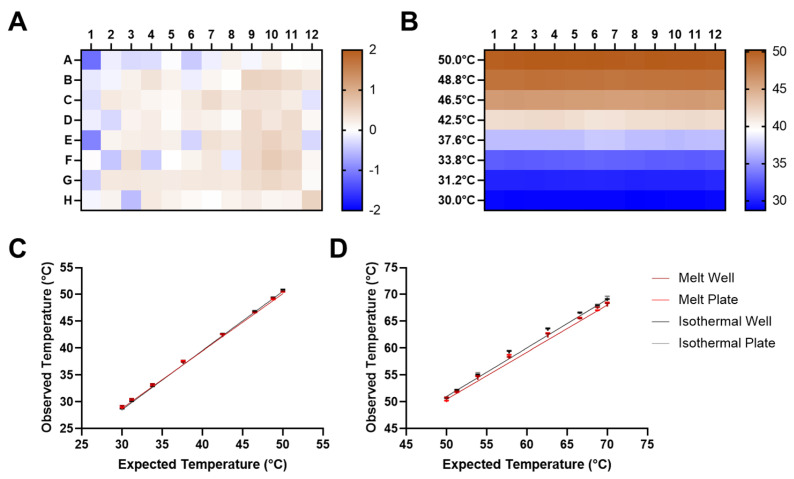
(**A**) Detection of non-uniform wells with an isothermal hold at 65 °C for 30 s. Relative temperature differences in individual wells were determined from deviation from the average of the isothermal standard curve for an entire 96-well plate (*n* = 96). The heat map indicates deviation from expected temperature in °C. (**B**) Temperature gradient uniformity in a 30–50 °C gradient held for 30 s. Temperature deviation in individual wells determined using the average of isothermal standard curves at specified temperatures for an entire 96-well plate (*n* = 8 × 12). Heat map indicates deviation from expected temperatures in each row (*n* = 12). Agreement between expected temperatures according to Bio-Rad CFX96 thermal cycler and observed temperatures obtained using standard curves from a 30–50 °C gradient (*n* = 8 × 12) (**C**) and a 50–70 °C gradient (*n* = 8 × 12) (**D**) with the same instrument. Melt Well: observed temperatures determined with standard curves from each individual well of an entire plate (melt curve run). Melt Plate: observed temperatures determined with the average standard curve from an entire plate (melt curve run). Isothermal Well: observed temperatures determined with standard curves from each individual well of an entire plate (isothermal run). Isothermal Plate: observed temperatures determined with the average standard curve from an entire plate (isothermal run). Error bars indicate standard deviation.

The DFPTB was tested in several applications that have utility for the calibration of real-time PCR thermocyclers, including block temperature uniformity in isothermal incubations and melt curve runs, as well as agreement between observed and expected temperatures across a temperature gradient. For the assessment of block temperature uniformity, the DFPTB was successful in detecting wells that deviated from the average across a 96-well plate set at 65 °C for 30 s (Figure 3A). Well discrepancy may be caused by either the presence of dust, corrosion or residues impeding heat transfer in the case of lower-than-expected temperatures as well as problems with evaporation due to seal failure in the case of higher-than-expected temperatures. The inclusion of the DFPTB in melt curve reaction samples using thermal cyclers with optical monitoring would enable real-time temperature quality control. For the assessment of block temperature gradient, the DFPTB was successfully applied to determine temperatures across a 96-well plate (Figure 3B). When a temperature gradient is programmed, thermal cyclers report expected temperatures for each row on the block. The DFPTB could measure deviations from the expected temperatures for gradients in the 30–50 °C (Figure 3C) and 50–70 °C ranges (Figure 3D).

For relative temperature determination in the physiological range, a minimum concentration of 125 nM DFPTB was required (Figure S3A). This was primarily due to the loss of mCherry fluorescence, which must be substantially above the background RFU for the determination of fluorescence ratios. For temperature determination in the 4–70 °C range, an mCherry signal-to-noise ratio of 2:1 is acceptable, corresponding to a concentration of 250 nM DFPTB (Figure S3A). The DFPTB could also identify deviations in well volume based upon the expected fluorescence ratio for the correct volume (Figure S3B).

Temperature deviations in PCR as low as 0.5 °C can result in incorrect or failing PCR results [32]. The ability to detect sub-degree deviations in well temperature in real-time with a simple and inexpensive probe is a valuable innovation. Our DFPTB with uvGFP increasing in fluorescence output and mCherry decreasing upon rising temperature (Figure S4) is likely to have superior sensitivity and resolution due to its improved dynamic range (i.e., the normalized fluorescence ratio increase over temperature will be steeper) than those with one stable fluorophore and the other decreasing or both decreasing in intensity [16].

**Figure 4:**
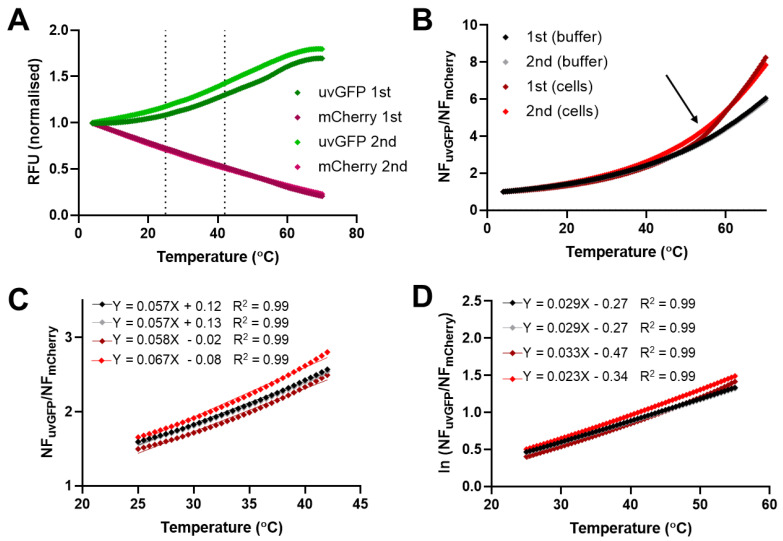
Fluorescence response of DFPTB in mixed *E. coli* bacteria compared to purified DFPTB in consecutive melt curve runs in PBS (pH 7.2). (**A**) Change in normalized fluorescence for uvGFP and mCherry. (**B**) Ratio of normalized fluorescence as a function of temperature. Arrow indicates likely bacterial cell lysis. (**C**) Pseudolinear range (25–42 °C). (**D**) Natural log-transformed ratio values in the range (4–55 °C). Vertical dotted lines: pseudolinear range.

The DFPTB works well with purified proteins. However, the purification of the fluorescent proteins is not essential as the DFPTB was successfully applied in a living bacteria-encapsulated format with mixed resuspended *E. coli* populations expressing mCherry and uvGFP (Figure 4, Figures S5 and S6). The response of the DFPTB to changing temperature in vivo is similar to that seen for the DFPTB in vitro in various buffers, both before and after cell lysis (Figure 4 and Figure S6), although with a more limited temperature range (Table 1). While the response of mCherry to different conditions is relatively stable, significant variations in uvGFP fluorescence response can be detected prior to and following cell lysis, particularly in extreme conditions (Figure S5). This is likely due to the dimerization of uvGFP at the intracellular concentrations present in the cells and subsequent transition to the monomeric form following cell lysis as well as its accelerated unfolding and loss of fluorescence at a lower temperature in low pH conditions. Here, the variation of DFPTB response between bacteria-encapsulated and lysed cell formats indicates its potential utility for the investigation of the permeability and the osmotic response of living cells to various buffer components [33].

Theoretically, the uvGFP and mCherry coding sequences could easily be engineered into a synthetic operon or to produce a fusion protein in order to facilitate the topologically precise measurement of absolute temperature. The latter could enable the single wavelength excitation of both fluorophores as GFP and mCherry are a well characterized fluorescence lifetime imaging microscopy (FLIM) fluorescence resonance energy transfer (FRET) pair [34,35], and the fluorescence lifetime of uvGFP is also sufficiently high for use as a FLIM-FRET-based thermosensor [36]. While real-time thermocyclers are not capable of measuring FLIM-FRET, the conversion of the DFPTB into a fusion protein would simplify its expression and purification and enable new or improved applications. As such, we envisage that a genetically encoded uvGFP-mCherry-based thermosensor may be superior to other examples where opposite fluorescence responses to temperature are not possible [10,14,15,37].

Most applications presented in this work have demonstrated the utility of the DFPTB to measure relative changes and deviations in temperature. However, the system could also be used to determine absolute temperature with instruments capable of measuring fluorescence intensity when coupled to an appropriate standard. For the calibration of real-time thermocyclers, the use of an in-well sensor with minimal measurement uncertainty would be required for the standardization of the DFPTB fluorescence ratio to absolute temperature [32]. While in-tube methods are not considered to be practical for routine use in metrology [32], they more truthfully record the environment experienced by a sample during typical use in thermocyclers [25,38].

In summary, the DFPTB developed in this work is a simple, cheap and sensitive biosensor for the reliable and reproducible measurement of relative change in temperature. The DFPTB demonstrated utility in several applications for real-time thermal cyclers, including the identification of non-uniformity in the heating block and evaluation temperature gradient accuracy. The DFPTB also demonstrated potential for future applications as a genetically encoded intracellular temperature sensor and for the topologically precise determination of absolute temperature.

## 4. Patents

Australian Provisional Patent No. 2023900341, Fluorescent protein temperature probe.

## Figures and Tables

**Table 1:** Ranges and sensitivities of the DFPTB in different buffer systems.

Buffer	pH	(NF_uvGFP_/NF_mCherry_)		ln(NF_uvGFP_/NF_mCherry_)	
		Range (°C)	Absolute Sensitivity (%°C^−1^)	Range (°C)	Absolute Sensitivity (%°C^−1^)
HS phosphate ^##^	7.8	25–42	6.7	4–70	3.0
Phosphate ^#^	7.8	25–42	6.0	4–75	2.8
PBS	7.2	25–42	5.7	4–70	2.7
PBS 1% BSA	7.2	25–42	6.0	4–75	2.6
PBS-T 4% BSA	7.2	25–42	5.6	4–75	2.6
HEPES *	7.5	25–42	5.2	4–70	2.6
Phosphate *	7.8	25–42	6.1	4–70	2.8
Phosphate	9.1	25–42	4.8	4–70	2.4
Phosphate	6.9	25–42	5.4	4–70	2.6
Phosphate	5.4	25–42	5.4	4–65	2.6
Phosphate	4.8	25–42	4.8	4–45	2.3
Citrate *	6.0	25–42	6.0	4–70	2.7
Bis-Tris *	6.0	25–42	5.4	4–60	2.5
Ammonium sulfate *	5.7	25–42	5.4	4–42	2.5
HS Phosphate ^##^^	7.8	25–42	5.9	4–55	2.8
Phosphate ^#^^	7.8	25–42	6.0	4–55	2.9
PBS ^^^	7.2	25–42	5.9	4–55	2.8
Phosphate ^^^	4.8	25–42	4.9	4–45	2.2

^#^ Phosphate buffer containing 300 mM NaCl, 10% glycerol, 20 mM imidazole and 2 mM β-mercaptoethanol; ^##^ same as phosphate ^#^ with 1.4 M NaCl; * 10% glycerol; ^ encapsulated *E. coli* highlighted in green. See methods for detailed buffer composition.

## Data Availability

The raw data presented in this study are available on request from the corresponding author.

## Supplementary Materials

The following supporting information can be downloaded at: https://www.mdpi.com/article/10.3390/bios13030338/s1, Table S1. Comparison of dual fluorescent thermosensors; Figure S1. (A) DFPTB ratio of normalized fluorescence fitted to an exponential (Malthusian) growth curve. (B) Changes in fluorescent protein ratio do not affect the DFPTB. (C) Incubation times between 1–60 s do not affect the DFPTB. (D) Resolution in the extended physiological range (25–55 °C) at 0.1 °C increments in continuous monitoring (melt curve). (E) Inset from (D) showing data for individual wells. (F) Inset from (D) with outlying well 1 data removed; Figure S2. Performance of the DFPTB in buffers with varying composition and pH; Figure S3. Effect on DFPTB dilution and detection of volume discrepancy. (A) Serial 2-fold dilutions of DFPTB in PBS, pH 7.2. (B) DFPTB aliquots of different volumes were performed in triplicate in PBS, pH 7.2; Figure S4. Absorbance and fluorescence spectra of uvGFP and mCherry at various temperatures. (A) uvGFP absorbance and (B) fluorescence spectra at 50 µM in PBS, pH 7.2. (C) mCherry absorbance and (D) fluorescence spectra at 2.5 µM in PBS, pH 7.2; Figure S5. Performance of DFPTB in *E. coli* prior to and following cell lysis; Figure S6. Performance of DFPTB in *E. coli* compared to purified DFPTB in various buffers. Refs. [14,15,18,19,20,21,22,39,40,41] are cited on the supplementary materials.

## References

[1] Zhu H., Zhang H., Xu Y., Lassakova S., Korabecna M., Neuzil P. (2020). PCR past, present and future. Biotechniques.

[2] Kunze M., Lattermann C., Diederichs S., Kroutil W., Buchs J. (2014). Minireactor-based high-throughput temperature profiling for the optimization of microbial and enzymatic processes. J. Biol. Eng..

[3] Du B., Zhang Z., Grubner S., Yurkovich J.T., Palsson B.O., Zielinski D.C. (2018). Temperature-Dependent Estimation of Gibbs Energies Using an Updated Group-Contribution Method. Biophys. J..

[4] Cianciulli C., Watzig H. (2011). Infrared-based temperature measurements in capillary electrophoresis. Electrophoresis.

[5] Lao A.I.K., Lee T.M.H., Hsing I., Ip N.Y. (2000). Precise temperature control of microfluidic chamber for gas and liquid phase reactions. Sens. Actuators A.

[6] McKenzie B.A., Grover W.H. (2017). A microfluidic thermometer: Precise temperature measurements in microliter- and nanoliter-scale volumes. PLoS ONE.

[7] Gill P., Moghadam T.T., Ranjbar B. (2010). Differential scanning calorimetry techniques: Applications in biology and nanoscience. J. Biomol. Tech..

[8] Wong F.H., Banks D.S., Abu-Arish A., Fradin C. (2007). A molecular thermometer based on fluorescent protein blinking. J. Am. Chem. Soc..

[9] Deepankumar K., Nadarajan S.P., Bae D.H., Baek K.H., Choi K.Y., Yun H. (2015). Temperature sensing using red fluorescent protein. Biotechnol. Bioprocess Eng..

[10] Savchuk O.A., Silvestre O.F., Adao R.M.R., Nieder J.B. (2019). GFP fluorescence peak fraction analysis based nanothermometer for the assessment of exothermal mitochondria activity in live cells. Sci. Rep..

[11] Ogle M.M., McWilliams A.D.S., Jiang B., Marti A.A. (2020). Latest trends in temperature sensing by molecular probes. ChemPhotoChem.

[12] Zhegalova N.G., Aydt A., Wang S.T., Berezin M.Y. (2013). Molecular thermometers for potential applications in thermal ablation procedures. Reporters, Markers, Dyes, Nanoparticles, and Molecular Probes for Biomedical Applications.

[13] Homma M., Takei Y., Murata A., Inoue T., Takeoka S. (2015). A ratiometric fluorescent molecular probe for visualisation of mitochondrial temperature in living cells. Chem. Commun..

[14] Nakano M., Arai Y., Kotera I., Okabe K., Kamei Y., Nagai T. (2017). Genetically encoded ratiometric fluorescent thermometer with wide range and rapid response. PLoS ONE.

[15] Lu K., Wazawa T., Sakamoto J., Vu C.Q., Nakano M., Kamei Y., Nagai T. (2022). Intracellular Heat Transfer and Thermal Property Revealed by Kilohertz Temperature Imaging with a Genetically Encoded Nanothermometer. Nano Lett..

[16] Feng G., Zhang H., Zhu X., Zhang J., Fang J. (2022). Fluorescence thermometers: Intermediation of fundamental temperature and light. Biomater. Sci..

[17] Liang S., Wang Y., Wu X., Chen M., Mu L., She G., Shi W. (2019). An ultrasensitive ratiometric fluorescent thermometer based on frustrated static excimers in the physiological temperature range. Chem. Commun..

[18] Zhang Z., Zhao Z., Wu L., Lu S., Ling S., Li G., Xu L., Ma L., Hou Y., Wang X. (2020). Emissive Platinum(II) Cages with Reverse Fluorescence Resonance Energy Transfer for Multiple Sensing. J. Am. Chem. Soc..

[19] Tang S., Wang N., Xu X., Feng S. (2019). A ratiometric fluorescent thermometer based on amphiphilic alkynylpyrene derivatives. N. J. Chem..

[20] Shi L., Song W., Lian C., Chen W., Mei J., Su J., Liu H., Tian H. (2018). Dual-emitting dihydrophenazines for highly sensitive and ratiometric thermometry over a wide temperature range. Anvanced Opt. Mater..

[21] Song W., Ye W., Shi L., Huang J., Zhang Z., Mei J., Su J., Tian H. (2020). Smart molecular butterfly: An ultrasensitive and range-tunable ratiometric thermometer based on dihydrophenazines. Mater. Horiz..

[22] Lv Y., Jin Y., Wu H., Liu D., Xiong G., Ju G., Chen L., Hu Y. (2019). An all-optical ratiometric thermometer based on reverse thermal response from interplay among diverse emission center and traps with high-temperature sensitivity. Ind. Eng. Chem. Res..

[23] Moreau M.J., Morin I., Schaeffer P.M. (2010). Quantitative determination of protein stability and ligand binding using a green fluorescent protein reporter system. Mol. Biosyst..

[24] Moreau M.J.J., Morin I., Askin S., Cooper A.E., Moreland N.J., Vasudevan S.G., Schaeffer P.M. (2012). Rapid determination of protein stability and ligand binding by differential scanning fluorimetry of GFP-tagged proteins. RSC Adv..

[25] Sanford L.N., Wittwer C.T. (2013). Monitoring temperature with fluorescence during real-time PCR and melting analysis. Anal. Biochem..

[26] Crews N., Ameel T., Wittwer C., Gale B. (2008). Flow-induced thermal effects on spatial DNA melting. Lab Chip.

[27] Erali M., Voelkerding K.V., Wittwer C.T. (2008). High resolution melting applications for clinical laboratory medicine. Exp. Mol. Pathol..

[28] Gao K., Oerlemans R., Groves M.R. (2020). Theory and applications of differential scanning fluorimetry in early-stage drug discovery. Biophys. Rev..

[29] Sorenson A.E., Schaeffer P.M. (2020). High-Throughput Differential Scanning Fluorimetry of GFP-Tagged Proteins. Methods Mol. Biol..

[30] Nagy A., Malnasi-Csizmadia A., Somogyi B., Lorinczy D. (2004). Thermal stability of chemically denatured green fluorescent protein (GFP): A preliminary study. Thermochim. Acta.

[31] Rana M.S., Wang X., Banerjee A. (2018). An Improved Strategy for Fluorescent Tagging of Membrane Proteins for Overexpression and Purification in Mammalian Cells. Biochemistry.

[32] Span M., Verblakt M., Hendrikx T. Comparison of temperature dynamics of various thermal cycler calibration methods. Proceedings of the 19th International Congress of Metrology.

[33] Wood J.M. (1999). Osmosensing by bacteria: Signals and membrane-based sensors. Microbiol. Mol. Biol. Rev..

[34] Soleja N., Manzoor O., Khan I., Ahmad A., Mohsin M. (2018). Role of green fluorescent proteins and their variants in development of FRET-based sensors. J. Biosci..

[35] Bajar B.T., Wang E.S., Zhang S., Lin M.Z., Chu J. (2016). A Guide to Fluorescent Protein FRET Pairs. Sensors.

[36] Spicer G., Efeyan A., Adam A.P., Thompson S.A. (2019). Universal guidelines for the conversion of proteins and dyes into functional nanothermometers. J. Biophotonics.

[37] Maksimov E.G., Yaroshevich I.A., Tsoraev G.V., Sluchanko N.N., Slutskaya E.A., Shamborant O.G., Bobik T.V., Friedrich T., Stepanov A.V. (2019). A genetically encoded fluorescent temperature sensor derived from the photoactive Orange Carotenoid Protein. Sci. Rep..

[38] Yang I., Kim Y.H., Byun J.Y., Park S.R. (2005). Use of multiplex polymerase chain reactions to indicate the accuracy of the annealing temperature of thermal cycling. Anal. Biochem..

[39] Ye F., Wu C., Jin Y., Chan Y.-H., Zhang X., Chiu D.T. (2011). Ratiometric temperature sensing with semiconducting polymer dots. J. Am. Chem. Soc..

[40] Tang J.-H., Sun Y., Gong Z.-L., Li Z.-Y., Zhou Z., Wang H., Li X., Saha M.L., Zhong Y.-W., Stang P.J. (2018). Temperature-Responsive Fluorescent Organoplatinum(II) Metallacycles. J. Am. Chem. Soc..

[41] Cui Y., Song R., Yu J., Liu M., Wang Z., Wu C., Yang Y., Wang Z., Chen B., Qian G. (2015). Dual-emitting MOF supersetdye composite for ratiometric temperature sensing. Adv. Mater..

