# Supplementary material for: Real-Time Temperature Sensing Using a Ratiometric Dual Fluorescent Protein Biosensor
Source: Biosensors (Basel). 2023 Mar 3;13(3):338. doi: 10.3390/bios13030338 (PMC10046200; doi:10.3390/bios13030338)
Supplement: Supplementary file 1 [file biosensors-13-00338-s001.zip › biosensors-2224306-supplementary-1.pdf]

biosensors

MDPI

Supporting Information

Real-time Temperature Sensing Using a Ratiometric Dual Fluorescent Protein Biosensor

Alanna E. Sorenson and Patrick M. Schaeffer *

Molecular and Cell Biology, College of Public Health, Medical and Veterinary Sciences, James Cook University, Douglas QLD 4811, Australia.

* Correspondence: Patrick.schaeffer@jcu.edu.au; Tel: +61 (0) 7 4781 4448

Table S1. Comparison of dual fluorescent thermosensors.

Material	Ratiometric Type	Temperature Range (°C)	Sensitivity (%° C^{-1})	Ref.
Polymer dot / rhodamine B	- ↓	10 – 70	0.23	[1]
Organoplatinum metallacycles	↓↓	−20 – 60	0.76	[2]
Multi-emission phosphors (Ce^{3+}/Mn^{2+})	↑↓	27 – 302	0.8	[3]
Europium MOF / perylene dye	- ↓	20 – 80	1.28	[4]
Amphiphilic alkynylpyrene derivatives	↑↓	0 – 80	< 1.0	[5]
B-gTEMP (mNeonGreen/tdTomat o)	↓↓	15 – 50	1.7	[6]
DFPTB ln(ratio)	↑↓	4 – 70	2.2 – 3.0	This work
gTEMP (Sirius/mT-Sapphire)	↓↓	5 – 50	2.6	[7]
Dual-emitting dihydrophenazines	↑↓	50 – 135	3.4	[8]
DFPTB (ratio)	↑↓	25 – 42	4.8 – 6.7	This work
Emissive platinum (II) cages	↑↓	−4 – 60	6.7	[9]
Dihydrophenzine derivatives	↑↓	18.4 – 37.7	140.1	[10]

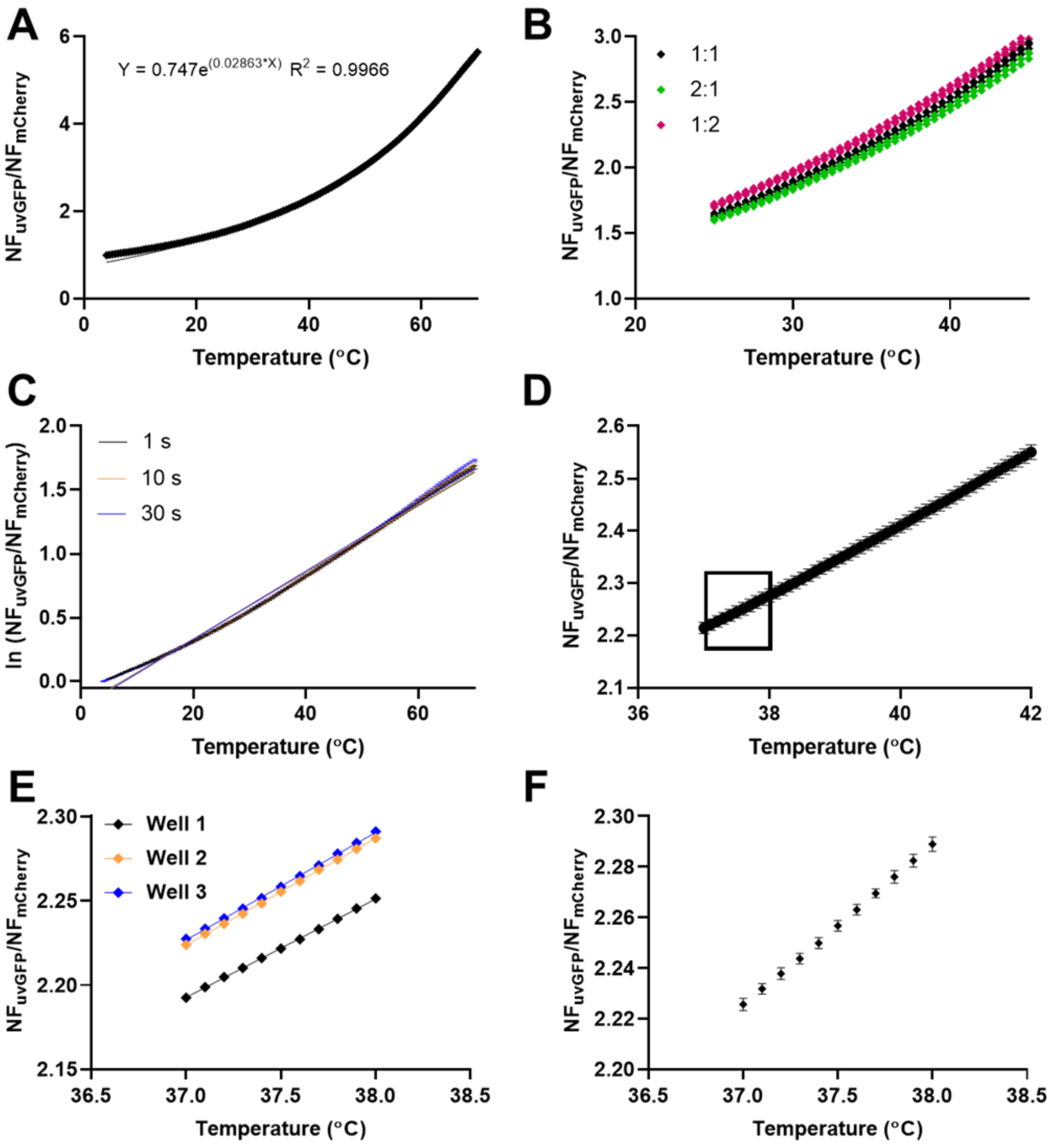

Figure S1. (A) DFPTB ratio of normalized fluorescence fitted to an exponential (Malthusian) growth curve. **(B)** Changes in fluorescent protein ratio do not affect the DFPTB. 1:1 = 1 μM each, 2:1 = 2 μM uvGFP and 1 μM mCherry, 1:2 = 1 μM uvGFP and 2 μM uvGFP. **(C)** Incubation times between 1-60 s do not affect the DFPTB. **(D)** Resolution in the physiological range (37–42 °C) at 0.1°C increments in continuous monitoring (melt curve). **(E)** Inset from **(D)** showing data for individual wells. **(F)** Inset from **(D)** with outlying well 1 data removed. Error bars indicate standard deviation. Data were analyzed using the Kruskal-Wallis test (P = 0.022). Pairwise comparison of changes in temperature between 0.2 °C increments were analyzed using paired t-tests (P values ranged from 0.003–0.0483).

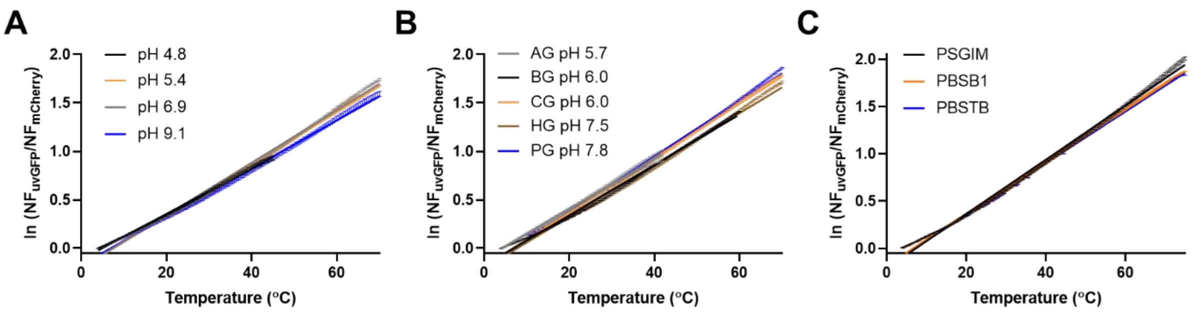

Figure S2. Performance of the DFPTB in buffers with varying composition and pH. **(A)** 50 mM phosphate buffer. **(B)** 50 mM buffers with 10% glycerol (G); A = ammonium sulphate, B = Bis-Tris, C = citrate, H = HEPES, P = phosphate. **(C)** Buffers with more complex composition; PSGIM = 50 mM phosphate, pH 7.8 with 10% glycerol, 300 mM NaCl, 20 mM imidazole, 2 mM β-mercaptoethanol, PBSB1 = phosphate buffered saline, pH 7.2 with 10% glycerol and 1% BSA, PBSTB = phosphate buffered saline, pH 7.2 with 0.005% Tween and 4% BSA. Error bars indicate standard deviation.

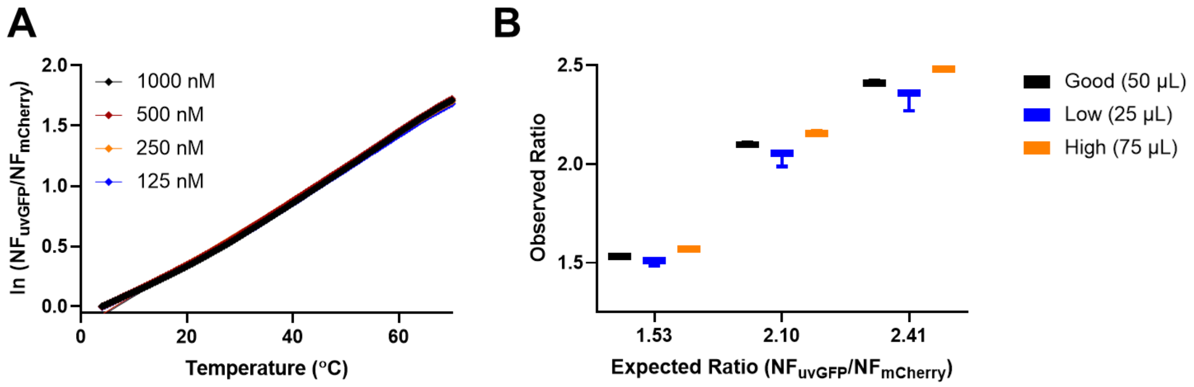

Figure S3. Effect on DFPTB dilution and detection of volume discrepancy. **(A)** Serial 2-fold dilutions of DFPTB in PBS, pH 7.2. **(B)** DFPTB aliquots of different volumes were performed in triplicate in PBS, pH 7.2. Agreement between expected ratio values for 50 µL aliquots of DFPTB and observed ratio values obtained using a standard 4–70 °C DSF run with aliquots of DFPTB at 25, 50 and 75 µL. Error bars indicate standard deviation.

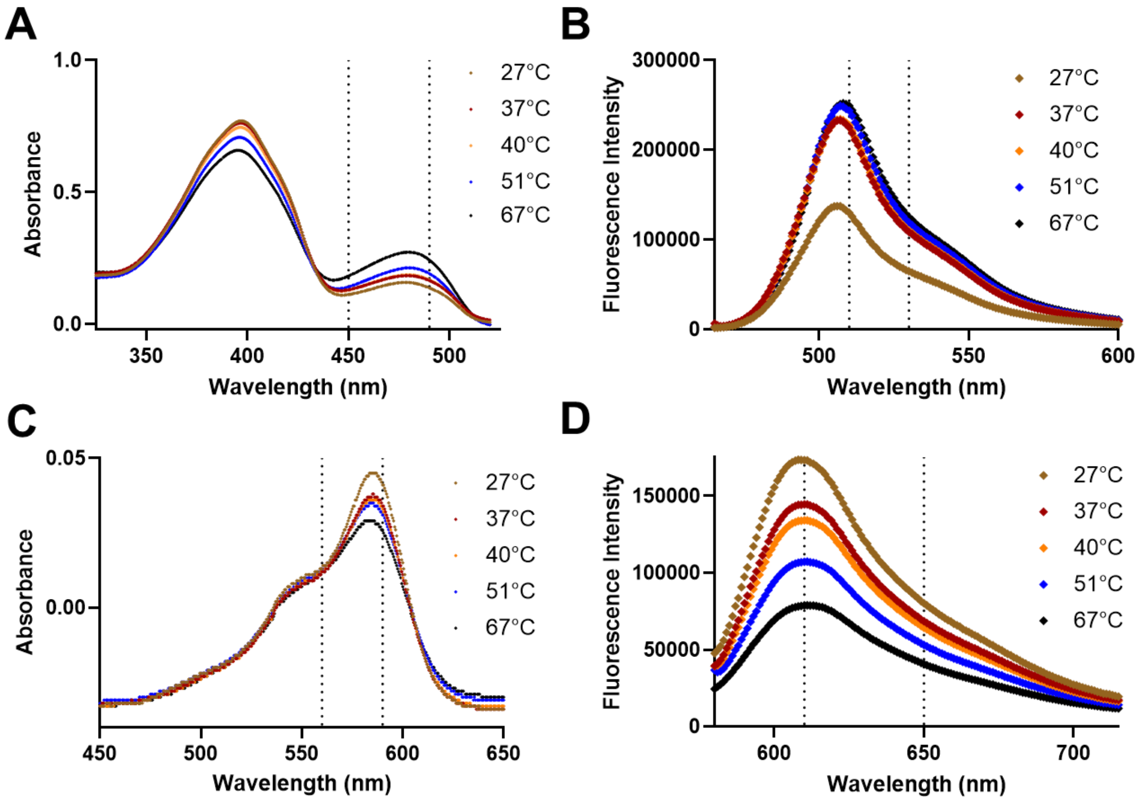

Figure S4. Absorbance and fluorescence spectra of uvGFP and mCherry at various temperatures**. (A)** uvGFP absorbance and **(B)** fluorescence spectra at 50 μM in PBS, pH 7.2. **(C)** mCherry absorbance and **(D)** fluorescence spectra at 2.5 μM in PBS, pH 7.2. Vertical dotted lines indicate excitation and emission wavelength ranges for Bio-Rad CFX96 FAM and Texas Red channels respectively.

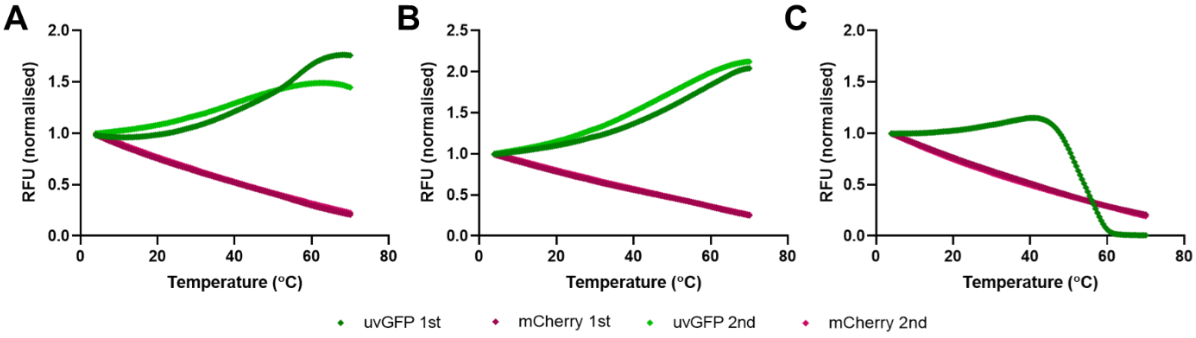

Figure S5. Performance of DFPTB in *E. coli* prior to and following cell lysis**. (A)** Lysis buffer (50 mM phosphate (pH 7.8), 300 mM NaCl, 10% glycerol, 20 mM imidazole, 2 mM β-mercaptoethanol). **(B)** High salt lysis buffer (lysis buffer with 1.4 M NaCl). **(C)** Low pH (50 mM phosphate, pH 4.8). uvGFP 1st and mCherry 1st: first melt curve run. uvGFP 2nd and mCherry 2nd: second melt curve run. No uvGFP data could be obtained in the second run at pH 4.8.

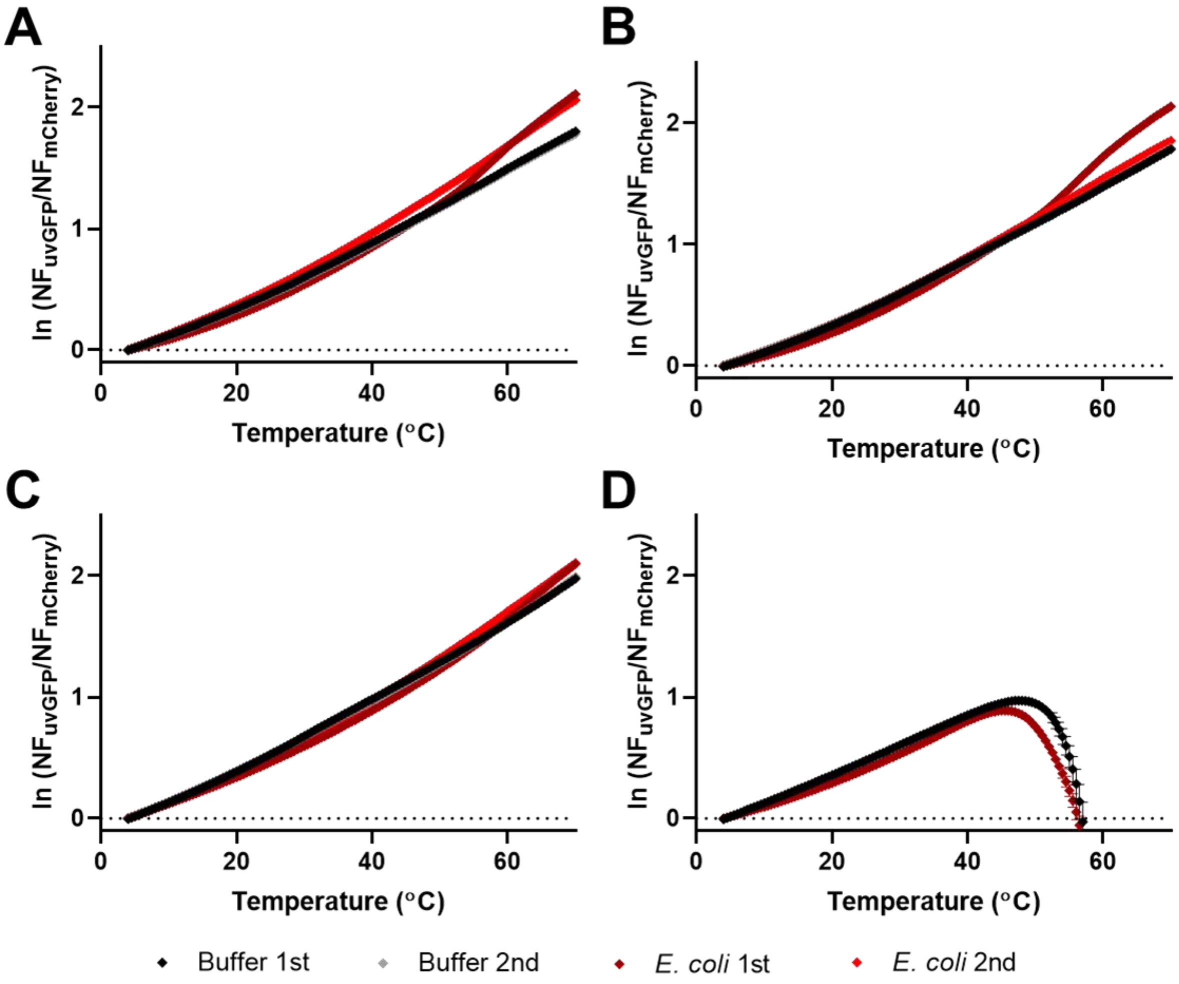

Figure S6. Performance of DFPTB in *E. coli* compared to purified DFPTB in various buffers**. (A)** PBS, pH 7.2. **(B)** Lysis buffer (50 mM phosphate (pH 7.8), 300 mM NaCl, 10% glycerol, 20 mM imidazole, 2 mM β-mercaptoethanol). **(C)** High salt lysis buffer (lysis buffer with 1.4 M NaCl). **(D)** Low pH (50 mM phosphate, pH 4.8). Buffer 1st and *E. coli* 1st: first melt curve run. Buffer 2nd and *E. coli* 2nd: second melt curve run. No second-round data are obtainable in low pH buffer as uvGFP is denatured in the first melt curve. Error bars indicate standard deviation.

References

1. Ye, F.; Wu, C.; Jin, Y.; Chan, Y.-H.; Zhang, X.; Chiu D.T. Ratiometric temperature sensing with semiconducting polymer dots. *J. Am. Chem. Soc.* **2011**, *133*, 8146–8149.
2. Tang, J.-H.; Sun, Y.; Gong, Z.-L.; Li, Z.-Y.; Zhou, Z.; Wang, H.; Li, X.; Saha, M.L.; Zhong, Y.-W.; Stang, P.J. Temperature-Responsive Fluorescent Organoplatinum(II) Metallacycles. *J. Am. Chem. Soc*. **2018**, *140*, 7723–7729.
3. Lv, Y.; Jin, Y.; Wu, H.; Liu, D.; Xiong, G.; Ju, G.; Chen, L.; Hu, Y. An all-optical ratiometric thermometer based on reverse thermal response from interplay among diverse emission center and traps with high-temperature sensitivity. *Ind. Eng. Chem. Res.* **2019**, *58*, 21242–21251.
4. Cui, Y.; Song, R.; Yu, J.; Liu, M.; Wang, Z.; Wu, C.; Yang, Y.; Wang, Z.; Chen, B.; Qian, G. Dual-emitting MOF supersetdye composite for ratiometric temperature sensing. *Adv. Mater.* **2015**, *27*, 1420–1425.
5. Tang, S.; Wang, N.; Xu, X.; Feng, S. A ratiometric fluorescent thermometer based on amphiphilic alkynylpyrene derivatives. *N. J. Chem.* **2019**, *43*, 6461–6464.
6. Lu, K.; Wazawa, T.; Sakamoto, J.; Vu, C.Q.; Nakano, M.; Kamei, Y.; Nagai, T. Intracellular Heat Transfer and Thermal Property Revealed by Kilohertz Temperature Imaging with a Genetically Encoded Nanothermometer. *Nano Lett.* **2022**, *22*, 5698–5707. https://doi.org/10.1021/acs.nanolett.2c00608.
7. Nakano, M.; Arai, Y.; Kotera, I.; Okabe, K.; Kamei, Y.; Nagai, T. Genetically encoded ratiometric fluorescent thermometer with wide range and rapid response. *PLoS ONE* **2017**, *12*, e0172344. https://doi.org/10.1371/journal.pone.0172344.
8. Shi, L.; Song, W.; Lian, C.; Chen, W.; Mei, J.; Su, J.; Liu, H.; Tian, H. Dual-emitting dihydrophenazines for highly sensitive and ratiometric thermometry over a wide temperature range. *Anvanced Opt. Mater.* **2018**, *6*, 1800190.

9. Zhang, Z.; Zhao, Z.; Wu, L.; Lu, S.; Ling, S.; Li, G.; Xu, L.; Ma, L.; Hou, Y.; Wang, X.; et al. Emissive Platinum(II) Cages with Reverse Fluorescence Resonance Energy Transfer for Multiple Sensing. *J. Am. Chem. Soc.* **2020**, *142*, 2592–2600. https://doi.org/10.1021/jacs.9b12689.
10. Song, W.; Ye, W.; Shi, L.; Huang, J.; Zhang, Z.; Mei, J.; Su, J.; Tian, H. Smart molecular butterfly: An ultrasensitive and range-tunable ratiometric thermometer based on dihydrophenazines. *Mater. Horiz.* **2020**, *7*, 615–623.
